# Juvenile moose stress and nutrition dynamics related to winter ticks, landscape characteristics, climate-mediated factors and survival

**DOI:** 10.1093/conphys/coab048

**Published:** 2021-07-07

**Authors:** Elias Rosenblatt, Jacob DeBow, Joshua Blouin, Therese Donovan, James Murdoch, Scott Creel, Will Rogers, Katherina Gieder, Nick Fortin, Cedric Alexander

**Affiliations:** 1 Vermont Cooperative Fish and Wildlife Unit, Rubenstein School of Environment and Natural Resources, University of Vermont, Burlington, VT 05405, USA; 2 US Geological Survey, Vermont Cooperative Fish and Wildlife Unit, Rubenstein School of Environment and Natural Resources, University of Vermont, Burlington, VT 05405, USA; 3 Rubenstein School of Environment and Natural Resources, University of Vermont, Burlington, VT 05405, USA; 4 Department of Ecology, Montana State University, Bozeman, MT 59717, USA; 5 Vermont Fish and Wildlife Department, Rutland, VT 05701, USA; 6 Vermont Fish and Wildlife Department, St. Johnsbury, VT 05819, USA

**Keywords:** Stress metabolites, nutritional restriction, non-invasive sampling, survival, moose, winter tick

## Abstract

Moose populations in the northeastern United States have declined over the past 15 years, primarily due to the impacts of winter ticks. Research efforts have focused on the effects of winter tick infestation on moose survival and reproduction, but stress and nutritional responses to ticks and other stressors remain understudied. We examined the influence of several environmental factors on moose calf stress hormone metabolite concentrations and nutritional restriction in Vermont, USA. We collected 407 fecal and 461 snow urine samples from 84 radio-collared moose calves in the winters of 2017–2019 (January–April) to measure fecal glucocorticoid metabolites (fGCM) concentrations and urea nitrogen:creatinine (UN:C) ratios. We used generalized mixed-effects models to evaluate the influence of individual condition, winter ticks, habitat, climate and human development on stress and nutrition in calf moose. We then used these physiological data to build generalized linear models to predict calf winter survival. Calf fGCM concentrations increased with nutritional restriction and snow depth during adult winter tick engorgement. Calf UN:C ratios increased in calves with lighter weights and higher tick loads in early winter. Calf UN:C ratios also increased in individuals with home ranges composed of little deciduous forests during adult winter tick engorgement. Our predictive models estimated that winter survival was negatively related to UN:C ratios and positively related to fGCM concentrations, particularly in early winter. By late March, as winter ticks are having their greatest toll and endogenous resources become depleted, we estimated a curvilinear relationship between fGCM concentrations and survival. Our results provide novel evidence linking moose calf stress and nutrition, a problematic parasite and challenging environment and winter survival. Our findings provide a baseline to support the development of non-invasive physiological monitoring for assessing environmental impacts on moose populations.

## Teaser

Lay Summary: We studied moose calves to investigate the links between stress and nutrition, a detrimental parasite during challenging winter conditions and winter survival. We identified several covariates related to fecal glucocorticoid metabolite concentrations and urea nitrogen:creatinine ratios and linked these physiological metrics to a survival rate.

## Introduction

Changing landscape conditions can exert pressures on wildlife populations, which are detrimental to the health and fitness of individuals (Angelier and Wingfield, 2013). Exposure to environmental stressors can activate an individual’s hypothalamic–pituitary–adrenal axis in a cascading process that releases glucocorticoid steroids (Wingfield, 2013). Glucocorticoid steroids, commonly referred to as stress hormones, can trigger physiological and behavioral responses that improve the chance of an individual surviving an immediate stressful event (Bonier *et al.*, 2009a, 2009b). However, prolonged elevation of glucocorticoid steroid concentrations can result in the suppression of reproduction, growth, immune function and responses to pathogens and parasites (Boonstra *et al.*, 1998; Sapolsky *et al.*, 2000). Ecological conditions can also induce nutritional restriction in wildlife, defined as the deficit in nutrients consumed relative to what is required for metabolic processes (DelGiudice, 1995). Prolonged nutritional strain causes individuals to exhaust stored fat reserves and catabolize muscle (DelGiudice *et al.*, 1987), leading to deterioration in body condition and consequential reduction in capacity to adapt to stochastic events (Parker, 2003). These physiological responses can have measurable impacts on an individual’s survival, reproductive success and offspring quality (Wingfield *et al.*, 1998; Meylan *et al.*, 2002; Parker, 2003; Evans *et al.*, 2006), with population-level implications (Angelier and Wingfield, 2013).

The monitoring of stress and nutrition in wildlife populations provides valuable insight into the physiological impacts of landscape conditions on wildlife populations (Von Der Ohe and Servheen, 2002; Millspaugh and Washburn, 2004; Romero, 2004; Garcia Pereira *et al.*, 2006). Glucocorticoid concentrations are an attractive metric to quantify the physiological response as they (or their metabolites) can be measured in blood, saliva, excrement or structures (Sheriff *et al.*, 2011). Nutritional restriction can be monitored through ratios of urea nitrogen to creatinine (UN:C) in urine deposited in snow (DelGiudice *et al.*, 1989). Increased UN:C ratios in animals with an otherwise low-protein diet reflect the catabolism of muscle tissue to meet protein and energy requirements as fat stores deplete, indicating nutritional restriction (DelGiudice, 1995). Chronic elevation of glucocorticoids and nutritional restriction in wildlife have been correlated with several factors of concern, including parasite loads (DelGiudice *et al.*, 1997; Raouf *et al.*, 2006; Ellingwood *et al.*, 2019), food availability (Moen and DelGiudice, 1997; Clinchy *et al.*, 2004; Kitaysky *et al.*, 2007), land use and development (Wasser *et al.*, 1997, 2011; Dantzer *et al.*, 2014), human activity (Creel *et al.*, 2002; Garcia Pereira *et al.*, 2006), predation pressure (Boonstra *et al.*, 1998; Christianson and Creel, 2010; Spong *et al.*, 2020), conspecific density (Saltz and White, 1991; Potratz *et al.*, 2019) and climate (Saltz and White, 1991; Wingfield *et al.*, 2018; Spong *et al.*, 2020).

As these physiological processes play pivotal roles in the survival of wild individuals, measures of stress hormone concentrations and nutritional restriction provide informative, low-cost indices of survival rates (DelGiudice, 1995; Escribano-Avila *et al.*, 2013). However, any relationship between these physiological metrics and survival are likely species- and context-specific. Studies across a variety of taxa have documented that stress hormone concentrations can be positively or negatively related to survival probability, likely due to species- and context-specific consequences of increased stress metabolite concentrations (Bonier *et al.*, 2009a). This relationship may instead be curvilinear, acknowledging the evolved benefits of stress for survival to an inflection point, where the further elevation of stress hormone concentrations becomes detrimental (Busch and Hayward, 2009). Nutritional restriction has been linked to decreased survival probability, yet this relationship may depend on risks taken by individuals to escape nutritional restriction (Villafuerte *et al.*, 1997) and may not be consistently detectable (Ellingwood *et al.*, 2019). Such uncertainties must be resolved if these physiological metrics are to be used to infer survival probabilities for free-ranging wildlife populations.

Moose (*Alces alces americana*) in the northeastern United States are ideal subjects to explore the links between environment, stress, nutrition and survival. In this region, the primary stressor of concern for moose is winter tick (*Dermacentor albipictus*), with rising infestations observed over the past 20 years (Musante *et al.*, 2010; Jones *et al.*, 2019; DeBow, 2020). With no behavioral or physiological adaptations to cope with winter ticks, moose can carry over 70 000 ticks in winter and spring months (December–April; Samuel 2004). During these months, moose are already nutritionally restricted until new, protein-rich vegetation begins to grow in spring (Ellingwood *et al.*, 2019). Winter tick infestations substantially increase a moose’s protein requirements as moose need to replenish blood lost to feeding winter ticks, compromising their ability to cope with already challenging winter conditions, particularly for smaller-bodied calves (Musante *et al.*, 2007). Winter tick infestations have increased winter mortality for moose calves and reduced reproductive success for adult moose, leading to regional population declines (Musante *et al.*, 2007, 2010; DeBow, 2020; Ellingwood *et al.*, 2020; Pekins, 2020).

Given regional population declines and the continued threat of winter tick infestations, studying stress and nutrition in eastern North American moose is critical for two reasons. First, modeling variation in fecal glucocorticoid metabolite (fGCM) concentrations (stress metric) and UN:C ratios (nutritional restriction metric) as a function of winter tick infestation and other limiting environmental variables would test for drivers of moose health and may elucidate avenues for management action. This insight is especially important as the moose is a game species throughout the region and legally managed by state and provincial wildlife authorities. Second, the development of non-invasive options for estimating vital rates using stress and nutrition metrics could benefit wildlife population management for species that are difficult and expensive to study. However, critical details remain, including the relationships between physiological metrics and survival, and when these signals are strongest and reliable for predictive purposes.

We investigated the connections between potential environmental challenges, stress metabolite and nutrition dynamics and survival rates of moose calves in Vermont, USA, from 2017 to 2019. Our study population was representative of many populations across the moose’s southern range in North America and characterized by recent population declines driven primarily by high winter tick infestations (Timmermann and Rodgers, 2017). We use repeated fecal and urine sampling from wild, radio-collared moose calves to assess the effects of an individual’s condition, winter tick engorgement, climate conditions, habitat composition and human development on (i) calf stress metabolite (fGCM) concentrations and (ii) calf nutritional restriction (UN:C ratios). We then (iii) developed predictive models to estimate calf survival rates using these nutrition and stress metrics. Our study explores the link between a stressor of paramount concern to variation in fGCM concentrations and UN:C ratios while identifying additional contributing environmental pressures. We also demonstrate the potential of using physiological metrics to estimate winter survival rates for a critical life stage of a long-lived species that is challenging to study.

## Methods

### Study area and moose population

**Figure 1:**
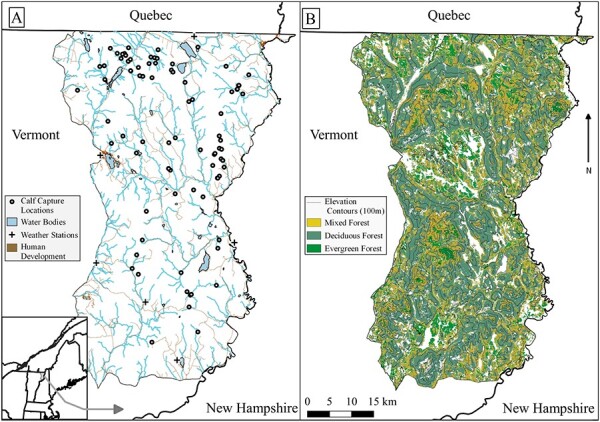
The study area for exploring connections between moose (*Alces alces*) calf (<1 year old) fGCM concentrations and UN:C ratios, winter tick infestation and survival in northeastern Vermont, USA, from 2017 to 2019. Moose were captured to be equipped with radio collars (white circles) and then followed for fecal and urine sample collection (locations not shown for clarity; **A**). Weather station data were collected at six locations across the study area. Forest types considered for habitat composition varied across the study area and by elevation (**B**).

We studied moose calf stress and nutrition in a ~1650 km^2^ area in northeastern Vermont, USA, within the state’s Wildlife Management Units E1 and E2 (N44.7778, W71.7520; Fig. 1; Vermont Department of Fish and Wildlife 2009). Vegetation in this region consisted of hardwood forests, mixed hardwood–conifer forests and conifer forests, with lowland conifer wetlands and bogs (Koitzsch, 2002). Forest stands varied in age across the area, due to historical and current timber harvest regimes (Koitzsch, 2002). Elevation ranged from 281 to 801 m above sea level across the study area (USGS, 2018). From 2017 to 2019, winter daily temperature ranged from −30.3°C to 18.6°C, with an average annual snowfall of 396 cm (NCDC, 2019). The area was a complex of large, publicly owned parcels managed for wildlife, fisheries and ecosystem conservation, large privately owned parcels used for timber and maple syrup production and small privately owned parcels with both exploitative and non-exploitative uses. Human activity in this area included timber harvest, maple sugar collection, snowmobile recreation, fishing, wildlife viewing, hunting and trapping.

The moose in this area represented Vermont’s core moose population and is a source of dispersing individuals for other Vermont and New England populations (Vermont Department of Fish and Wildlife 2009). Moose were not limited by predation pressure or food availability but instead by parasites and regulated harvest (Vermont Department of Fish and Wildlife, 2009; DeBow, 2020). Historically, this area contained the highest moose density in the state, with a peak of 1.67 moose/km^2^ in 2005 (Vermont Department of Fish and Wildlife, 2020). Moose densities in the study area and across the state have since decreased, initially with increased legal harvests aimed to reduce population densities to meet management goals (~0.68 moose/km^2^; Vermont Department of Fish and Wildlife 2009). Population densities declined past management goals despite reductions in legal harvests due to reduced calf survival and adult female reproductive rates, primarily driven by winter ticks (Vermont Department of Fish and Wildlife, 2020). The moose density estimate in our study area during the final year of this study was 0.67 moose/km^2^ [80% confidence interval (CI): 0.61–0.77 moose/km^2^; Vermont Department of Fish and Wildlife 2020], a 60% reduction from the density estimated in 2005 (C. Alexander, unpublished data). Even with these changes, the study area contained an estimated 50% of Vermont’s moose, despite comprising only 8.5% of Vermont’s land area (Vermont Department of Fish and Wildlife 2020).

### Animal capture and radio collaring

We focused our study on 90 radio-collared moose calves (<1 year old; *n*_female_ = 38, *n*_male_ = 52) captured in January of 2017–2019 (30 animals captured each year). With radio-collared animals, we could repeatedly sample individuals and consider variables of interest that would not be available without individual recognition. We captured moose using aerial net-gunning by helicopter and physical restraint (Carpenter and Innes, 1995). Drug immobilization was used in rare instances when net-gunning was not feasible. Once restrained, capture personnel equipped the animal with an expandable Survey Globalstar V7.1 GPS collar (Vectronic Aerospace GmbH, Germany) that emitted a VHF signal and transmitted GPS coordinates every 13 hours. Collars had a mortality switch that would activate after 5 hours of inactivity. All animal handling protocols were approved by the University of Vermont’s Institution Animal Care and Use Committee (protocol #17-035).

### Fecal and urine sample collection and lab analysis

We measured fGCM concentrations and UN:C ratios from radio-collared calves using non-invasive, repeated fecal and urine sampling across three winters (2017–2019). These physiological metrics are commonly used as proxies for chronic stress and nutritional restriction, respectively (DelGiudice, 1995; Dantzer *et al.*, 2014; Sheriff *et al.*, 2011). Winter sampling was ideal for the objectives of this study as cold ambient temperatures preserve steroid metabolites and proteins in freshly deposited feces and urine, respectively. Restricting sampling to winter also minimized the potential confounding effects of variation in diet, territoriality, breeding and social interactions on fGCM concentrations and UN:C ratios (Parker, 2003; Millspaugh and Washburn, 2004).

We attempted to collect fecal samples and urine samples from radio-collared calves every 2 weeks from 20 January to 7 April during each year of the study (six possible sampling occasions per year). We collected samples from radio-collared calves only in their first year of life. We waited at least 7 days after capture to begin fecal sampling to avoid measuring elevated fGCM due to capture and handling (Dehnhard *et al.*, 2001; Hämäläinen *et al.*, 2014). We used collar GPS locations, VHF radio-telemetry, direct observation and tracking to collect a random sample of 5–7 fresh fecal pellets from fecal piles and 10 ml of fresh urine from each defecation site located. Fecal pellets and urine from calves were distinguished from their mother’s using the sizes of tracks and pellets. Samples were collected with a rubber glove to minimize contamination and placed in a Whirl-Pak (Nasco, Wisconsin, USA) then stored at −20°C until overnight shipment for lab steroid and protein extraction, where samples were stored at −80°C. Annual fecal and urine sampling ceased before the loss of winter snowpack in early to mid-April.

Fecal samples were thawed, individually homogenized, subsampled (1.5 g) and then dried in a rotary evaporator for 6 hours. Steroid metabolites were extracted by boiling ~0.2 g of dried material in 10 ml of 95% ethanol. The supernatant was dried and reconstituted in 1 ml of 95% methanol. Enzyme-linked immunosorbent assays were used to quantify fGCM concentrations (Enzo Life Sciences ADI-900-071). Procedural validation established that antibody displacement was parallel for moose fecal extracts and known standards and that recovery of known standards was accurate. A 31-fold dilution of fecal extracts was found to maximize precision and intra- and inter-assay coefficients of variation were 9.9% and 14.4%, respectively. We detected no association between fGCM and the water content (*r*^2^ = 0.023) or mass of indigestible material (*r*^2^ = 0.007) in fecal samples and therefore did not include these covariates in subsequent analysis. Samples were assayed in duplicate, and those with a coefficient of variation above 20% were re-assayed. We divided the dilution-corrected mean (ng/ml) fGCM concentration by the mass of extracted dry material to yield ng fGCM/g of dry feces for each sample. Concentrations were log_e_-transformed to normalize variance.

We melted collected urine samples and subsampled 1 ml of urine for lab analysis. Samples were refrozen at −20°C and sent to Biovet, Inc. (Barneveld, Wisconsin, USA) for measurement of urea nitrogen (UN) and creatinine (C; mg/dl), consistent with procedures reported by Ellingwood et al. (2019). These data were expressed as a ratio (UN:C) and these ratios were also log_e_-transformed to normalize variance.

### Explanatory variables of fGCM concentrations and UN:C ratios (objectives 1 and 2)

Despite the lack of predators and an abundance of suitable forage throughout our study area, winter ticks exacerbated the challenges posed by winter conditions. While calves were accompanied by their mothers during this period, calves were weaned by early fall and relied on their physical condition to survive challenging winter months with low-nutrition forage typical for moose (Musante et al., 2007). Questing larval winter ticks attached to calves (and all other moose) from vegetation during fall months (September–October) and progressed through nymph and adult life stages throughout the winter, taking progressively larger blood meals (Samuel 2004). This exacerbation has led to reduced calf survival rates (Vermont Department of Fish and Wildlife, 2009; DeBow, 2020) and likely resulted in stress responses and nutritional restriction (as measured by fGCM concentrations and UN:C ratios, respectively).

We measured multiple variables with potential influence on stress and nutritional restriction, grouped by individual condition, winter tick engorgement, climate conditions, habitat composition and human development hypotheses (Table 1). Individual condition in early winter, particularly heavy weights and low parasite loads, may reduce fGCM concentrations and UN:C ratios in moose calves. Individual characteristics were recorded before sampling during each January capture. We recorded the sex of each individual and measured their weight using the capture helicopter’s internal load scale. We then measured their relative winter tick load by summing the number of ticks on four 10-cm transects on both the rump and shoulder and measured lungworm (*Dictyocaulus* sp.) abundance from fecal samples, using the McMasters flotation technique (University of Maine Animal Health Laboratory, Orono, Maine, USA).

**Table 1:** Variables considered to explain patterns of stress (S) and nutrition (N) in moose (*Alces alces*) calves (<1 year old) in northeastern Vermont, USA. Some of these variables were also considered as transformed or cumulative variables (see Methods)

Variable (unit)	Hypothesis	Sampling source	Variable range	Model set	References
Year	Null	Observation	2017, 2018, 2019	S, N	Dantzer *et al.*, 2016
Sex	Individual condition	Capture	Female, male	S, N	Millspaugh and Washburn, 2004; Romero, 2004
Weight	Individual condition	Capture	109–231 kg	S, N	Musante *et al.*, 2007
Tick load	Individual condition	Capture	0–100 ticks	S, N	Jones *et al.*, 2017; Zohdy *et al.*, 2017
Lungworm load	Individual condition	Capture	0–133 eggs	S, N	Debow, 2020
Nutritional restriction (UN:C)	Winter tick engorgement	Observation	0.12–20.8 mg/dl	S	Ellingwood *et al.*, 2019; Palme, 2019
Engorged adult female winter tick (percent ticks engorged)	Winter tick engorgement	Observation	0–22%	S, N	Drew and Samuel, 1989
Maximum and minimum weekly temperatures	Climate conditions	National Climate Data Center (2019)	−8.6 to 7.9°C	S, N	Renecker and Hudson, 1986
Snow depth	Climate conditions	National Climate Data Center (2019)	9.9–75.9 cm	S, N	Coady, 1974
Mixed forest in home range	Habitat composition	National Land Cover Dataset (2019)	9.4–45.7%	S, N	Wasser *et al.*, 2011
Deciduous forest in home range	Habitat composition	National Land Cover Dataset (2019)	0.4–70%	S, N	Wasser *et al.*, 2011
Conifer forest in home range	Habitat composition	National Land Cover Dataset (2019)	0.7–45.6%	S, N	Wasser *et al.*, 2011
Forage structure (0–3 m) in home range	Habitat composition	Lidar	14.1–27.5%	S, N	Blouin *et al.*, 2021
Human development in home range	Human development	Vermont Center for Geographic Information (2019)	0.0–5.1%	S	Millspaugh *et al.*, 2001
Snowmobile trail density in home range	Human development	Vermont Center for Geographic Information (2019)	0.0–4.5%	S	Creel *et al.*, 2002

Winter tick engorgement likely has a great impact on moose calf stress physiology and nutrition, given the additional protein demands calves incur from this infestation (Musante *et al.*, 2007). Engorged adult female ticks take the greatest blood meal relative to other life stages and exert the greatest toll on moose (Samuel, 2004). As the winter progresses, nymphs molt into their adult form and begin to engorge starting in February. By early April, the proportion of engorged adult females reaches its peak and rapidly decreases as adult females drop off their hosts to deposit their eggs (Samuel, 2004). For each week of our winter sampling season, we estimated the percentage of winter ticks carried by moose that were engorged adult females using Drew and Samuel’s (1989) model of winter tick development. This winter tick development model quantified the distribution of life stages for winter ticks found on a moose throughout a winter, which we verified with winter tick infestation data from recovered calves in a co-occurring survival study (Debow, 2020). We considered this metric of percent ticks engorged both as untransformed and inverse (1/engorgement) in our model selection, allowing for linear or asymptotic relationships with stress metabolite concentrations and nutritional restriction. We also considered cumulative engorgement through time, as a measurement of the cumulative impact of winter tick engorgement throughout a winter. For stress models, we also considered nutritional restriction (UN:C ratios) for each individual as a measure of nutritional stress (presumed to be largely influenced by winter tick engorgement), from fresh urine samples collected alongside fecal samples as previously described.

Climate conditions such as snowpack depth and warm winter temperatures (> −5°C) have been documented to increase the energetic costs for calves, reducing their ability to move and forage across a landscape (Coady, 1974; Renecker and Hudson, 1986). We incorporated measurements of snow depth, changes in snow depth and maximum temperatures in our explanatory models of fGCM concentrations and UN:C ratios. We measured winter snowpack by averaging weekly snow depths from six weather stations on the study site (NCDC 2019; Fig. 1) during each sampling occasion. We considered mean snow depth during each sampling occasion and 2 weeks prior, and the change in snow depth between sampling occasions. We also averaged weekly maximum temperatures recorded at these six stations.

We measured habitat composition within each animal’s home range to account for the influence of suitable winter cover and forage habitat in our models of fGCM concentrations and UN:C ratios. We used 95% fixed kernel density estimates to delineate each animal’s home range during each winter (January–April) of the study period using collar-collected GPS locations and the *kernelUD()* and *getverticesHR()* functions in the adehabitatHR package in R (Calenge, 2006; R Core Team, 2020). We used data from the 2016 National Land Cover Dataset (USGS, 2019) at a 30 m^2^ buffered to 1 km to quantify the proportion of coniferous, deciduous and mixed forest types in each animal’s home range (Koitzsch, 2002; Yang *et al.*, 2018). We also used light detection and ranging (lidar) data collected in November 2016 at a 10 m^2^ resolution buffered to 1 km to calculate the proportion of vegetation in an animal’s home range that was <3 m tall, which represented potential forage for the radio-collared moose (Moen *et al.*, 1998; USGS, 2016). These 1-km buffers acted as smoothers for both land cover and structure data to summarize what, on average, is available to a moose in its home range.

Finally, we considered human development as a contributor to moose fGCM concentrations, as these areas may elicit avoidance behavior to humans and vehicles (Frid and Dill, 2002; Baker *et al.*, 2013). We estimated the proportion of each individual’s home range covered with impervious surfaces from the 2016 National Land Cover Dataset (USGS, 2019), buffered to 1 km. We also estimated the proportion covered with Vermont Association of Snow Travelers (VAST) trails, as snowmobiles have been linked to stress responses (Creel *et al.*, 2002; VCGI, 2019).

### Analytical methods

For objectives 1 and 2, we fit sets of candidate generalized linear mixed models explaining the variation in moose calf fGCM concentrations (objective 1) and UN:C ratios (objective 2). We fit models using the *lmer*() function in the lme4 package (Bates *et al.*, 2015) and included a random effect of individuals to account for heterogeneity and potential lack of independence between repeated measures (Coppes *et al.*, 2018). We dropped highly correlated (*r*_pearson_ > 0.6) covariates and tested for temporal autocorrelation using the *acf()* function in R (R Core Team, 2020). We dropped outlier fGCM and UN:C measurements that were identified as potential influential points using the *cooksdistance()* function in the stats package (R Core Team, 2020).

We used a three-stage process for fitting and refining our explanatory models. First, we considered univariate and additive combinations of variables *within* each hypothesis (Table 1). All models included year as a categorical fixed effect. We used Akaike’s information criterion (Akaike, 1998) corrected for small sample size (AICc) to identify the best-supported, most parsimonious (delta AICc <2) models for each hypothesis (null, individual condition, winter tick engorgement, climate conditions, habitat composition and human development models). This preliminary analysis allowed us to identify those models that best represented each hypothesis.

Second, we compared top supported models *across* hypotheses using AICc scores to infer the relative support of each hypothesis to other, competing hypotheses. We used AICc weights of these top classification models to infer the relative support across hypotheses. This analysis allowed us to rank our hypotheses and identify which hypotheses were best supported by our stress and nutrition data.

Third, we evaluated explanatory models that included the effects of multiple hypotheses. We fit additive combinations of hypothesis models that were better supported than the null model (delta AICc_hypothesis_ − delta AICc_null_ ≤ −2), along with interactions between percent ticks engorged and snow depth, and percent ticks engorged and habitat composition (if present in hypothesis models). We used these interactions to test the hypotheses that (i) physiological effects of percent ticks engorged and snow depth are dependent on each other and that (ii) home ranges composed of a greater proportion of certain forest cover types could reduce physiological impacts from ticks during peak engorgement by providing beneficial cover from winter weather (Timmermann and McNicol, 1988). We did not test any other interaction as we wanted to minimize the risk of overfitting our explanatory models. We determined the best performing explanatory model(s) using AICc for interpreting the primary drivers of winter fGCM concentrations and UN:C ratios in moose. If multiple models received substantial support (∆AICc <2), we calculated model-averaged coefficient estimates using the AICcmodavg package (Mazerolle, 2020) in R for our inferences.

For objective 3, we examined the ability to use winter fGCM concentrations and UN:C ratios to predict calf survival. We documented mortality and cause of death for perished calves with comprehensive necropsies within 24 hours of death. We only considered mortality for calves that perished from natural causes after 20 January (the start of our sampling season) until 15 May of each year (when sufficient forage exists for animals to recover). Our data were insufficient to fit binomial mixed-effect generalized linear models, so we partitioned the data into six, 2-week datasets for our analysis (late January, early February, late February, early March, late March, early April).

We fit binomial generalized linear models (glm) to estimate the relationship of fGCM and UN:C (independent variables) to calf winter survival (dependent variable) for each 2-week dataset using the glm() function in R (R Core Team, 2020). We used AICc model selection to test whether calf winter survival was related to fGCM and UN:C ratios. We considered both linear and quadratic effects of fGCM concentrations (Busch and Hayward, 2009) and linear effects of UN:C ratios and included calf sex given reported differences in winter survival rates (DeBow, 2020). We did not account for annual variation in survival as our goal was to create a predictive survival model that could be applied in future years. We then compared these 2-week predictive models to identify when sampling would best predict survival. For this model comparison, we calculated mean fGCM and UN:C values for each 2-week period to impute missing data for calves that were not sampled during each period.

## Results

**Figure 2:**
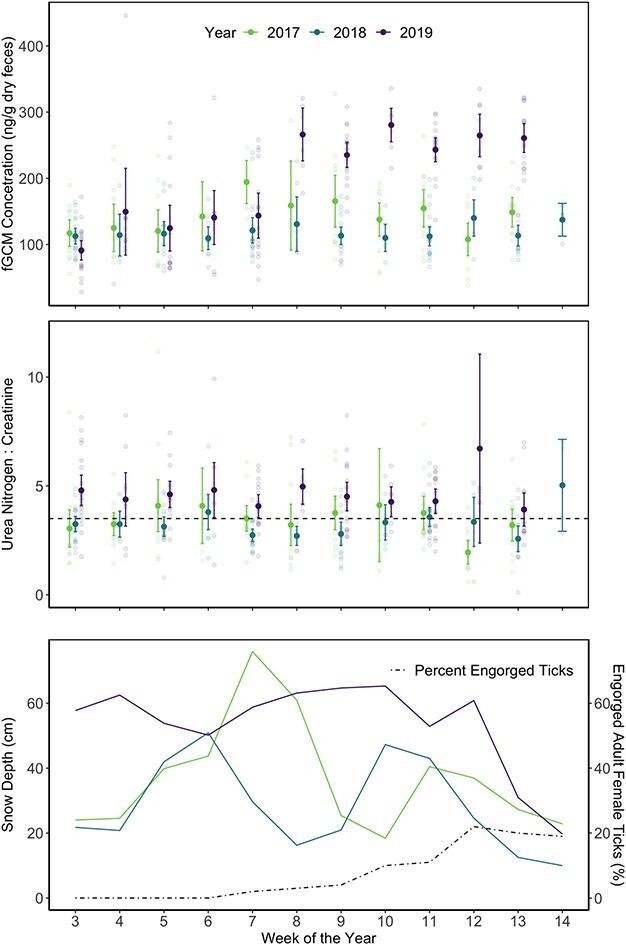
Distribution of (**A**) stress metabolite concentrations (fGCM), (**B**) urea nitrogen:creatinine (UN:C) ratios from radio-collared moose calves and (**C**) the temporal variation in snow depth (colored lines) and percent ticks engorged (dashed line; Drew and Samuel, 1989). Mean and 95% confidence limits are indicated with dark circles and error bars, with light circles representing measurements from moose calves. Weeks indicate sampling occasions across the winter season. Four samples exceeded a UN:C ratio of 10 and were not shown in this figure.

We collected and processed 407 fecal samples and 461 urine samples from 84 radio-collared moose calves (Fig. 2A and B). The remaining radio-collared calves (*n* = 6) were not accessible for repeated sampling. Each calf was sampled an average of 4.8 times in a winter (range, one to six occasions). Over half of these calves (*n* = 43) perished as a result of winter tick infestations, other parasites and various natural causes (DeBow, 2020), mostly after urine and fecal sampling had concluded in early April (*n* = 30). fGCM concentrations ranged from 28.07 to 445.93 (ng/g dry feces; mean = 149.11, SD = 70.4; Fig. 2A). UN:C ratios ranged from 0.12 to 24.4 (mean = 3.74, SD = 2.01; Fig. 2B). Annual variation in snow depths was apparent, with 2019 maintaining deeper snowpack relative to 2017 and 2018, despite a spike in snow depth in 2017 of over 70 cm (Fig. 2C). Duration of deep winter snowpack also varied by year, overlapping with the increase of engorged female winter ticks on moose in March and early April (weeks 9–14; Fig. 2C; Drew and Samuel 1989).

### Objective 1: drivers of moose stress metabolite concentrations

The first step of our three-stage model selection process identified the best-supported combination of variables *within* each hypothesis. Model selection results identifying the best-supported hypothesis models explaining stress metabolite concentrations are presented in the Supplemental Materials (Table S1). Our best individual condition model included calf sex only (in addition to year, which was included in all competing models). The best-supported tick engorgement model included UN:C ratios and inverse percent ticks engorged. The top climate condition model accounted for snow depth 2 weeks before samples were taken, and the maximum temperature in the week a sample was taken. Our best-supported habitat composition model incorporated the proportions of a calf’s home range composed of deciduous and mixed forests. The best human development model included the proportion of a calf’s home range composed of developed areas.

Our second step identified which stress hypotheses were best supported by comparing these top models. Our stress hypothesis model comparison indicated that winter tick engorgement was strongly supported relative to other hypotheses (Table 2). Our hypothesis that stress was driven by individual condition was not well supported (∆AICc was within 2 AICc scores of the null hypothesis model’s ∆AICc) and was not included in our multi-hypothesis model comparisons (Step 3; Table 2).

**Table 2:** Hypothesis comparison for stress and nutrition for moose calves in northeastern Vermont, USA. Models included random effects for individual moose and were identified as best supported additive combinations of variables for each hypothesis (Table S1). Models that were supported more than the null model (>2 delta AICc from null model) were considered in constructing multi-hypothesis explanatory models of stress and nutrition

Dataset	Hypothesis	Model	K	ΔAICc	Weight
Moose calf stress	Stress is driven by winter tick engorgement	log(fGCM) ~ year + (1/percent ticks engorged) + UN:C	6	0	1
	Stress is driven by climate conditions	log(fGCM) ~ year + prior snow depth + maximum temperature	6	37.92	0
	Stress is driven by habitat composition	log(fGCM) ~ year + deciduous forest + mixed forest	6	69.46	0
	Stress is driven by human activity	log(fGCM) ~ year + development	5	72.59	0
	Stress is driven by individual condition	log(fGCM) ~ year + sex	5	75.41	0
	Null	log(fGCM) ~ year	4	76.35	0
Moose calf nutrition	Nutrition is driven by habitat composition	log(UNC) ~ year + mixed forest + deciduous forest	6	0	0.975
	Nutrition is driven by individual condition	log(UNC) ~ year + weight + tick load	6	7.39	0.024
	Nutrition is driven by winter tick engorgement	log(UNC) ~ year + percent ticks engorged	5	15.12	0.001
	Nutrition is driven by climate conditions	log(UNC) ~ year + current snow depth	5	15.73	0
	Null	log(UNC) ~ year	4	17.98	0

**Figure 3:**
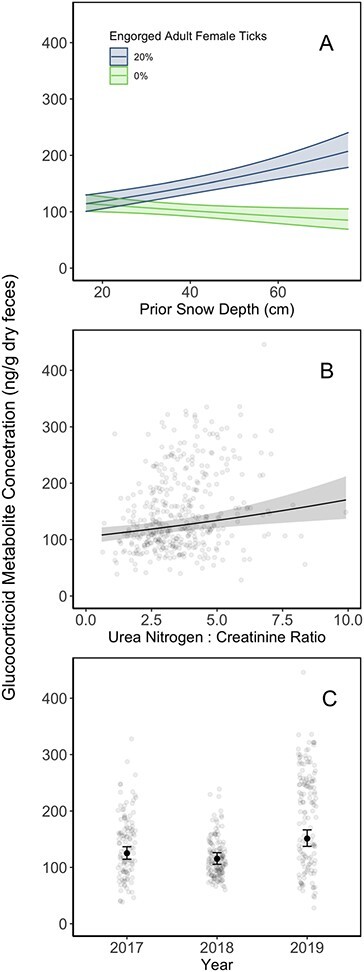
Estimated effects of variables model-averaged across the top stress metabolite explanatory models for radio-collared moose calves in northeastern Vermont. Average fGCM concentrations increased with snow depth during periods of female winter tick engorgement (**A**) and with the animal’s UN:C ratio (**B**) and was highest during the 2019 study season (relative to 2017 and 2018 seasons; **C**).

Our third and final step identified an explanatory model(s) of calf stress metabolite concentrations that could include variables from multiple hypotheses. Our best-supported stress models included winter tick engorgement and climate conditions hypotheses with an interaction between percent ticks engorged and snow depth two weeks prior, with varying support for the inclusion of habitat composition and human development (Table 3; Fig. 3). We used model averaging across the top three models (<2 ∆AICc) for our inferences (Table 4). Calf fGCM concentrations were elevated in 2019 but did not significantly differ between 2017 and 2018 (Table 4; Fig. 3). Inverse percent ticks engorged, UN:C ratios and snow depth from 2 weeks prior in a calf’s home range were positively related to calf fGCM concentrations (Table 4; Fig. 3). The interaction between percent ticks engorged and snow depth 2 weeks prior resulted in elevated stress response during high engorgement, high snow depth periods and reduced stress response during low engorgement periods (Table 4; Fig. 3). Model-averaged 95% CIs for maximum weekly temperature, human development and deciduous and mixed forest compositions did not differ from zero (Table 4).

**Table 3:** Model selection results for additive combination of supported hypotheses (Table 2) for stress and nutrition dynamics for moose in northeastern Vermont, USA. Models included random effects for individual moose. Interactions (along with their main effects) were considered between percent ticks engorged and snow depth, and percent ticks engorged and habitat composition (indicated with : ). For each model set, we model average coefficient estimates from models within 2 ∆AICc of the best-supported model. Competing models within 10 ∆AICc are shown, with null models listed for reference. Prior snow depth refers to average snow depth 2 weeks before sampling

Stress models	K	ΔAICc	Weight
log(fGCM) ~ year + (1/percent ticks engorged) : prior snow depth + UN:C + prior snow depth + maximum temperature + mixed forest + deciduous forest	11	0	0.37
log(fGCM) ~ year + (1/percent ticks engorged) : prior snow depth + UN:C + maximum temperature + development + mixed forest + deciduous forest	12	0.27	0.33
log(fGCM) ~ year + (1/percent ticks engorged) : prior snow depth + UN:C + maximum temperature + development	10	0.63	0.27
log(fGCM) ~ year + (1/percent ticks engorged) : prior snow depth + UN:C + prior snow depth + maximum temperature	9	5.35	0.03
log(fGCM) ~ year	4	121.96	0
			
Nutrition models	K	ΔAICc	Weight
log(UNC) ~ year + weight + tick load + percent ticks engorged : deciduous forest + current snow depth + mixed forest	11	0	0.582
log(UNC) ~ year + weight + tick load + percent ticks engorged : deciduous forest + mixed forest	10	0.76	0.399
log(UNC) ~ year + weight + tick load + percent ticks engorged : mixed forest + current snow depth + deciduous forest	11	7.98	0.011
log(UNC) ~ year + weight + tick load + percent ticks engorged : mixed forest + deciduous forest	10	9.00	0.006
log(UNC) ~ year	4	48.59	0

**Table 4:** Model-averaged coefficient estimates with standard errors (SE) and 95% confidence intervals (CIs) from the best supported models of stress and nutrition identified in Table 3. Coefficients were estimated using log_e_-transformed fGCM concentrations and UN:C ratios

	Calf stress (fGCM)		Calf nutrition (UN:C)	
Variable	Estimate (SE)	95% CI	Estimate (SE)	95% CI
Intercept	4.139 (0.167)	3.812–4.465	1.300 (0.219)	0.871–1.728
Year—2018	−0.082 (0.062)	−0.204—0.041	−0.038 (0.053)	−0.141 to 0.066
Year—2019	0.19 (0.069)	0.054–0.326	0.277 (0.057)	0.165–0.388
Weight	—	—	−0.003 (0.001)	−0.005 to −0.001
Tick load	—	—	0.004 (0.001)	0.001–0.006
(1/percent ticks engorged)	0.259 (0.112)	0.04–0.478	—	—
Percent ticks engorged	—	—	0.019 (0.006)	0.008–0.031
UNC	0.049 (0.015)	0.019–0.079	—	—
Snow depth 2 weeks prior	0.011 (0.002)	0.007–0.014	—	—
Current snow depth	—	—	0.001 (0.001)	−0.002 to 0.004
Maximum weekly temperature	0.002 (0.005)	−0.008 to 0.012	—	—
Human development	0.003 (0.003)	−0.003 to 0.009	—	—
Percent deciduous forest	0.006 (0.004)	−0.003 to 0.014	−0.0003 (0.0016)	−0.004 to 0.003
Percent mixed forest	0.006 (0.005)	−0.004 to 0.016	0.006 (0.005)	−0.004 to 0.016
(1/percent ticks engorged) : prior snow depth	−0.016 (0.003)	−0.021 to −0.01	—	—
Percent ticks engorged : percent deciduous forest	—	—	−0.001 (0.0001)	−0.0008 to −0.0003

### Objective 2: drivers of moose UN:C ratios

Our results here followed the same three-stage model selection process as detailed in the methods and objective 1 results.

*Step 1—*Model selection results identifying the best-supported hypothesis models explaining UN:C ratios are presented in the Supplemental Materials (Table S1). Our top individual condition model included tick load and weight at capture (before sampling). The best tick engorgement model included percent ticks engorged. The top climate condition model accounted for average snow depth when a sample was taken. Our best-supported habitat composition model incorporated the proportions of a calf’s home range composed of deciduous and mixed forests. Our measure of conifer forest composition of home ranges was strongly negatively correlated with deciduous forests (*r*_pearson_ = −0.8), so we could not include these two variables in the same UN:C ratio model.

*Step 2—*Habitat composition was the best-supported hypothesis explaining variation in UN:C ratios for calves (Table 2). The individual condition hypothesis carried 2.4% of the model weight; all other hypotheses received no support (≤0.1% of the model set weight). All top hypothesis models were better supported than the null hypothesis (delta AICc scores were not within 2AICc of the null hypothesis model’s delta AICc score) and were included in our multi-hypothesis model comparisons (Step 3; Table 2).

**Figure 4:**
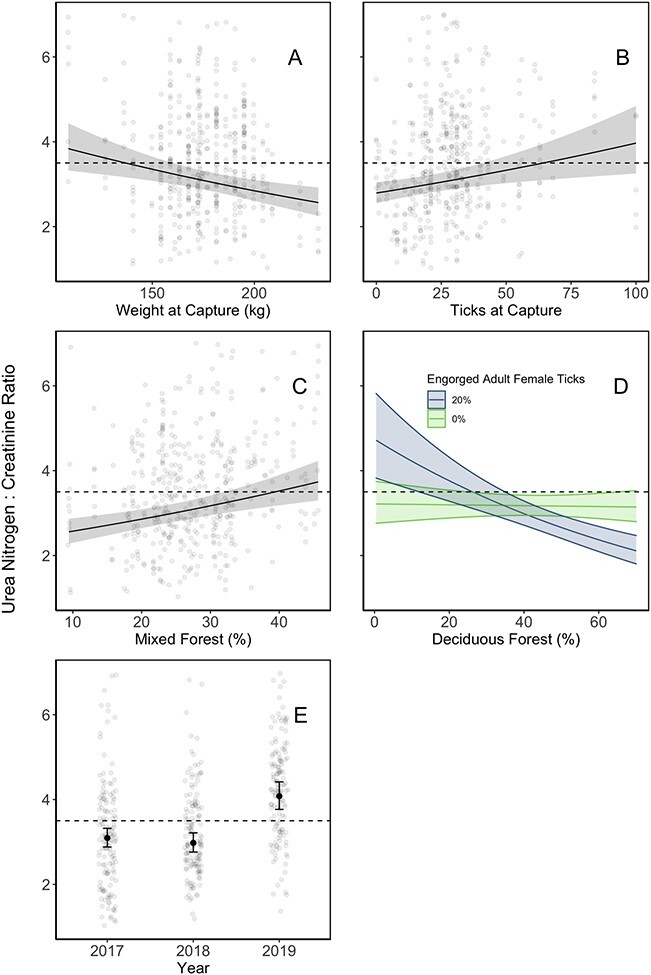
Estimated effects of variables model-averaged across the top nutrition explanatory models for radio-collared moose calves in northeastern Vermont. The threshold between normal and severe winter nutritional restriction (UN:C = 3.5) is indicated with a horizontal dashed line. Average UN:C ratios decreased with higher calf weight and increased with higher tick load at the beginning of the study season (January; **A** and **B**, respectively). Average UN:C ratios were related to habitat composition in an animal’s home range, particularly during peak adult winter tick engorgement (**C** and **D**). Average UN:C ratios were highest during the 2019 field season, relative to 2017 and 2018 seasons (**E**).

*Step 3—*Our top calf UN:C ratio models (< 2∆AICc) both included individual condition, winter tick engorgement and habitat composition hypotheses, with an interaction between tick engorgement and habitat composition variables (Tables 3). The climate condition hypothesis (i.e. snow depth) was also incorporated in one of these top models (Table 3). We again used model averaging across these two models for our inferences (Table 4). On average, UN:C ratios were high in 2019, compared to 2017 and 2018 (Table 4; Fig. 4). Calves with lower weights and higher winter tick loads at capture in mid-winter (early January) were associated with higher UN:C ratios throughout the winter (Table 4; Fig. 4). As percent ticks engorged increased throughout the sampling season calf UN:C ratios would increase, and this deterioration was influenced by habitat composition (Table 4; Fig. 4). Calf UN:C ratios was positively related to the proportion of a home range composed of mixed forest but was negatively related to the proportion of deciduous forest available when more adult female winter ticks become engorged (Table 4; Fig. 4). Model-averaged 95% CIs for the effect of snow depth during sampling did not differ from zero (Table 4).

### Objective 3: predictive models of calf survival using stress and nutrition

**Figure 5:**
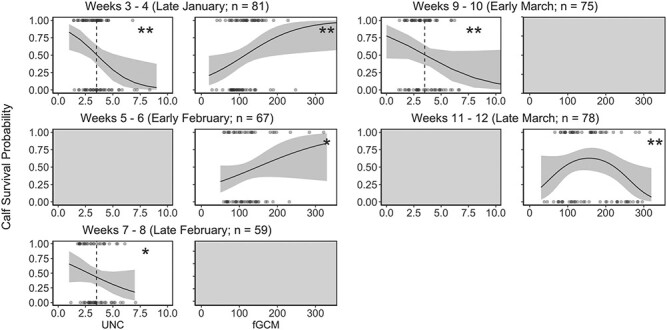
Predicted relationships of nutrition and stress to calf survival using samples collected in six, 2-week intervals. Estimated relationships between predictors with 85% and 95% confidence are indicated (^*^ and ^**^, respectively). In UN:C plots, the threshold between normal and severe winter nutritional restriction (UN:C = 3.5) is indicated with a vertical dashed line.

Our predictive models for calf survival indicated sampling periods when stress and nutrition metrics may aid in estimating calf winter survival (Tables 5 and 6; Fig. 5). Overall, fGCM concentrations were positively related to survival (though this shifted later in winter), and UN:C ratios were negatively related to survival. However, the strength of these relationships varied by the sampling period. In late January, the top predictive model of survival included a negative role of UN:C ratios and a positive role of fGCM, with 95% confidence for these estimated roles (Table 6; Fig. 5). In February, fGCM and UN:C had weakening roles, with best-supported models for early and late February including only fGCM or UN:C, respectively, both with estimated effects with 85% confidence (Table 6; Fig. 5). In early March, the negative role of UN:C ratios in predicting survival continued (with 95% confidence; Table 6; Fig. 5). By late March, our top model included a quadratic relationship between fGCM and survival but did not include UN:C ratios (Table 6; Fig. 5). By early April, the null model was the best-supported model (Table 5).

**Table 5:** Model selection results of predictive models of calf survival using fGCM concentrations and UN:C ratios, while considering sex and year. Data were partitioned into 2-week intervals throughout the sampling season. Linear and polynomial relationships were considered for fGCM concentrations, whereas only a linear relationship was considered for UN:C. The best-supported model was used for interpretation, though these models were not always supported over the null intercept-only model

Sampling occasion	Model	K	ΔAICc	Weight
Weeks 3–4 (late January)	Survival ~ sex + fGCM + UNC	4	0	0.656
	Survival ~ sex + fGCM + fGCM^2^ + UNC	5	2.16	0.223
	Survival ~ sex + UNC	3	4.68	0.063
	Survival ~ sex + fGCM	3	5.63	0.039
	Survival ~ sex + fGCM + fGCM^2^	4	7.29	0.017
	Survival ~ 1	1	13.09	0.001
Weeks 5–6 (early February)	Survival ~ sex + fGCM	3	0	0.354
	Survival ~ sex + fGCM + UNC	4	1.1	0.204
	Survival ~ sex + fGCM + fGCM^2^	4	1.59	0.16
	Survival ~ sex + UNC	3	2.07	0.126
	Survival ~ sex + fGCM + fGCM^2^ + UNC	5	2.62	0.095
	Survival ~ 1	1	3.51	0.061
Weeks 7–8 (late February)	Survival ~ sex + UNC	3	0	0.467
	Survival ~ sex + fGCM + UNC	4	2.28	0.149
	Survival ~ sex + fGCM	3	2.35	0.144
	Survival ~ 1	1	2.4	0.141
	Survival ~ sex + fGCM + fGCM^2^ + UNC	5	4.47	0.05
	Survival ~ sex + fGCM + fGCM^2^	4	4.51	0.049
				
Weeks 9–10 (early March)	Survival ~ sex + UNC	3	0	0.565
	Survival ~ sex + fGCM + UNC	4	2.19	0.189
	Survival ~ sex + fGCM + fGCM^2^ + UNC	5	3.4	0.103
	Survival ~ sex + fGCM	3	3.73	0.088
	Survival ~ sex + fGCM + fGCM^2^	4	5.43	0.037
	Survival ~ 1	1	7.05	0.017
Weeks 11–12 (late March)	Survival ~ sex + fGCM + fGCM^2^	4	0	0.412
	Survival ~ sex + fGCM	3	1.95	0.155
	Survival ~ sex + fGCM + fGCM^2^ + UNC	5	2.21	0.137
	Survival ~ sex + UNC	3	2.23	0.135
	Survival ~ 1	1	2.71	0.106
	Survival ~ sex + fGCM + UNC	4	4	0.056
Weeks 13–14 (early April)	Survival ~ 1	1	0	0.358
	Survival ~ sex + fGCM	3	0.44	0.288
	Survival ~ sex + UNC	3	2.35	0.111
	Survival ~ sex + fGCM + UNC	4	2.35	0.111
	Survival ~ sex + fGCM + fGCM^2^	4	2.69	0.093
	Survival ~ sex + fGCM + fGCM^2^ + UNC	5	4.46	0.039

**Table 6:** Coefficient estimates for best-supported logistic generalized linear regression models predicting calf survival, by 2-week periods. Estimates, standard errors (SE) and 95% confidence intervals (CIs) that deviate from zero are indicated in bold. Estimates with 85% CIs that deviate from zero are also indicated (*)

	Weeks 3–4 (late January)		Weeks 5–6 (early February)		Weeks 7–8 (late February)	
Variable	Estimate (SE)	95% CI	Estimate (SE)	95% CI	Estimate (SE)	95% CI
Intercept	1.42 (1.12)	−0.77 to 3.64	−0.48 (0.75)	−1.98 to 0.98	1.78 (1.02)	−0.09 to 3.99
Sex—male	**−1.51 (0.56)**	**−2.65 to −0.46**	**−1.41 (0.58)**	**−2.62 to −0.30**	**−1.28 (0.58)**	**−2.48 to −0.17**
fGCM	**0.014 (0.006)**	**0.003–0.028**	0.009 (0.006)	−0.002 to 0.022*	—	—
fGCM^2^	—	—	—	—	—	—
UN:C	**−0.62 (0.26)**	**−1.19 to −0.17**	—	—	−0.36 (0.24)	−0.87 to 0.09*
						
	Weeks 9–10 (early March)		Weeks 11–12 (late March)			
Variable	Estimate (SE)	95% CI	Estimate (SE)	95% CI		
Intercept	2.08 (0.87)	0.55–3.94	−1.66 (1.57)	−4.87 to 1.36		
Sex—male	**−1.49 (0.55)**	**−2.62 to −0.45**	**−1.12 (0.52)**	**−2.19 to −0.13**		
fGCM	—	—	0.04 (0.02)	−0.002 to 0.080*		
fGCM^2^	—	—	**−0.0001 (0.0001)**	**−0.0002 to −0.0001**		
UN:C	**−0.35 (0.20)**	**−0.76 to −0.01**	—	—		

We compared these predictive models across sampling periods to identify when fGCM concentrations and UN:C ratios could best estimate winter calf survival rates. After imputing missing fGCM and UN:C values with biweekly means, our late January (weeks 3–4) model that included the roles of both fGCM concentrations and UN:C ratios received the most support out of any model from any sampling occasion (Table 7). All late January candidate models received 88% of the cumulative model weight (Table 7). No other model using both stress metabolite concentration and UN:C ratio received any support (≥1% of the model set weight). These results indicate that late January sampling is optimal for monitoring winter calf survival rates in northeastern Vermont.

**Table 7:** Model comparison results for models predicting survival. Missing data were imputed using 2-week means to allow comparison of models across 2-week sampling occasions. Only models that performed better than a model predicting survival by sex are shown. Sample occasion indicates the data used in each candidate model

Model	Sampling occasion	K	ΔAICc	Weight
Survival ~ sex + fGCM + UNC	Late January	4	0	0.585
Survival ~ sex + fGCM + fGCM^2^ + UNC	Late January	5	2.18	0.197
Survival ~ sex + UNC	Late January	3	4.72	0.055
Survival ~ sex + fGCM	Late January	3	5.76	0.033
Survival ~ sex + UNC	Early March	3	7.23	0.016
Survival ~ sex + fGCM	Early April	3	7.37	0.015
Survival ~ sex + fGCM + fGCM^2^	Late January	4	7.53	0.014
Survival ~ sex + fGCM	Early February	3	8.61	0.008
Survival ~ sex + UNC	Late February	3	8.83	0.007
Survival ~ sex + fGCM + UNC	Early April	4	8.96	0.007
Survival ~ sex + fGCM + fGCM^2^	Late March	4	9.09	0.006
Survival ~ sex	NA	2	9.15	0.006

## Discussion

Moose calves in this study exhibited shifts in both stress metabolite concentrations and UN:C ratios related to various environmental factors. Our models indicated that fGCM concentrations and UN:C ratios were not only impacted by winter tick infestation (the primary stressor of concern and driver of population declines), but also by climate, habitat and individual condition. We also documented relationships between survival and these physiological metrics during discrete windows in our study, informing the use of these metrics for non-invasive monitoring of vital rates. By repeatedly sampling known individuals, we could account for inherent physiological differences between individuals and variables detailing individual conditions and spatial use (Dantzer *et al.*, 2014). Our design controlled for any differences in fGCM concentration and UN:C ratios across seasons, changes in nutrition and life events (i.e. pregnancy) as our study subjects were all calves, sampled in the same season of the year when forage is relatively poor. Our inferences inform our understanding of how this critical age class responds to the compounding challenges of winter and a parasite responsible for regional population decline. We note that our study was correlative, and future research may benefit from an experimental design focused on isolating the influence of winter ticks from other responses to the environment.

Biases from diurnal patterns in stress, delayed stress responses to environmental cues and high-protein forage elevating UN:C ratios (instead of nutritional restriction) are likely minimal given our study species and ecosystem. Though our study design could not control for diurnal patterns in stress metabolite concentrations (Millspaugh and Washburn, 2004; Palme, 2019), these patterns likely do not impact our inferences because moose have long digestion times (21.3 hours for rumen turnover time; Hjeljord et al. 1982) and excrete elevated metabolites in feces 12–36 hours after an induced acute stress response (Crouse, 2003; Thompson *et al.*, 2020). Further, we estimated spatial covariates for each year of the study and temporal covariates weekly (Thompson et al. 2020) to accommodate this delay in stress responses to the environment. Finally, we could not test if elevated UN:C ratios indicate that moose are nutritionally restricted (and catabolizing muscle tissue) or accessing protein-rich food sources (Saltz *et al.*, 1995). However, we find the latter scenario unlikely, as available winter forage for moose is very low in protein and no supplemental feeding program exists (DelGiudice, 1995; DelGiudice *et al.*, 1997). While specific to our sampled population in northeastern Vermont, our findings provide insight into the dynamic nature of how moose calves physiologically respond to multiple environmental stressors.

Winter tick epizootics, characterized by >50% parasite-induced winter calf mortality, are a driving factor for moose population dynamics in the region (Murray *et al.*, 2006; Jones *et al.*, 2019; DeBow, 2020). We expected stress and nutritional responses to winter tick infestation and engorgement, as moose have no evolved behavior to escape the increasing toll of attached winter ticks throughout winter and early spring months (i.e. grooming strategies; Samuel, 2004). Stress metabolite concentrations increased with the percent of attached winter ticks that were engorged adult females and with the calf’s level of nutritional restriction (as measured by UN:C ratio). We estimated that UN:C ratios were influenced by the percentage of ticks that were engorged and tick load measured at capture at the beginning of each sampling season. In concert, winter ticks were responsible for much of the elevated stress metabolite concentrations and nutritional restriction during a time when protein-rich forage is scarce, which in turn compounds a calf’s stress response.

With no known behaviors to counter tick attachment or engorgement, moose calves could benefit from conditions that reduce winter stress and nutritional restriction. First, calves experienced lower UN:C ratios (and therefore lower stress metabolite concentrations) if they were heavier and carried fewer ticks at the beginning of winter. Calves of greater weight in early winter are better prepared to survive both ‘normal’ winter challenges (e.g. snowpack, poor quality food) and the added metabolic costs of winter tick infestation (Musante *et al.*, 2007). Calf weight is determined largely by their mother’s condition during the previous winter (Keech *et al.*, 2000), as the majority of fetal development occurs in the final trimester, which coincides with peak adult winter tick engorgement (1 March to 16 May; Pekins 2020). Mothers in poor condition tend to produce lighter calves and give birth later in the birthing season (early May to late June; Keech *et al*., 2000), diminishing the calf’s ability to enter winter with suitable body mass (Keech *et al.*, 1999). Tick attachment to calves is a separate process, largely determined by climate conditions when ticks are not attached to their host and by the distribution of optimal forage and localized high densities of their host (Blouin *et al.*, 2021; Samuel, 2004; Dunfey-Ball, 2017; Healy *et al.*, 2018, 2020).

Second, moose calves can avoid some of the physiological detriment of winter tick engorgement when there is less snow on the ground during peak engorgement. Snow depth is not usually considered a limitation for moose in the northeastern United States, as sustained depths rarely exceed thresholds known to impede movement and contribute to mortality (60 cm and 90 cm, respectively; Coady 1974). We estimated a positive relationship between stress metabolite concentrations and snow depth 2 weeks before sampling as adult winter ticks became engorged. Unlike in 2017 and 2018, snow depth in 2019 remained consistently above 50 cm well into peak adult engorgement. This severe winter contributed to this estimated relationship during peak winter tick engorgement, with average stress metabolite concentrations during this period exceeding averages from any other time of this study. Notably, fGCM concentrations decreased with increasing snow depth when no engorged adult females were present on moose. This trend is likely an artifact of low snow depths rapidly increasing during this period of no tick engorgement in 2017 and 2018. Our results indicate that the duration and depth of the snowpack compound the physiological toll of winter tick engorgement.

Finally, moose calves can benefit when their home ranges are composed of habitat components that reduce their nutritional restriction and stress metabolite concentrations. During winter months moose utilize conifer stands as protection from deepening snow and cold weather while foraging on recently grown woody material from deciduous and coniferous species (Timmermann and McNicol, 1988). Our models estimated that mixed forest cover was associated with increased UN:C ratios in moose calves, while deciduous forest cover was related to decreased UN:C ratios as more ticks became engorged. We also infer that coniferous forest cover was associated with increased UN:C ratios given a high negative correlation between coniferous and deciduous forests in our nutritional restriction analysis. These results are likely due to abiotic conditions correlated with these broad forest cover classifications. There is likely no meaningful difference in forage quality in our measures of habitat composition as winter forage is so poor in quality that moose cannot consume enough forage to meet their nutritional requirements, even in the absence of winter tick infestation (Schwartz and Renecker, 2007; Pekins, 2020). The deciduous forests in our study area occupy lower elevation areas, whereas mixed forests and evergreen forests generally occupy mid- to high-elevation areas, respectively. Moose persisting in higher elevation areas would incur energetic costs with greater slope angles and deeper snow depths that persist longer. Winters with deep, persistent snowpack across our study area (e.g. 2019) would exacerbate these energetic costs at higher elevations and therefore in mixed and conifer forests. Our study indicates the potential importance of low elevation, deciduous forests for moose calves coping with winter tick infestations during challenging winter conditions.

Notably, we did not detect physiological responses to exposure to human development in our study. We observed only low proportions of human development (mean = 1.3%, max = 5.1%) and snowmobile trails (mean = 1.2%, max = 4.5%) in animal home ranges. Much of the human development in the interior of our study area included only access roads for seasonal homes and activities such as timber harvest. Despite sharing their home ranges with humans, at these low levels of development, moose may be able to limit associated risk effects from humans. However, this may change if human development in this area intensifies.

A common criticism of studies describing physiological metrics in wild populations is that the links between these metrics and a meaningful vital rate or fitness surrogate are often assumed, but not investigated (Walker *et al.*, 2005; Wikelski and Cooke, 2006; Bonier *et al.*, 2009a). We estimated that stress metabolite concentrations were positively associated with winter survival probability from late January through early February, while our nutrition metric was negatively associated with survival in late January, late February and early March. Stress responses in long-lived organisms evolved to allocate energy to survive in challenging environmental conditions (Angelier and Wingfield, 2013; Wingfield, 2013). However, moose calves that do not have the endogenous resources to overcome environmental stressors may exhibit low fecal metabolite concentrations (Busch and Hayward, 2009), confirmed by the negative relationship of UN:C ratios with winter survival rate. These relationships were estimated early in our study season before the metabolic toll of winter tick engorgement and months of deteriorating body condition. Calves in poorer condition exhibit low stress metabolite concentrations and high UN:C ratios during these early winter months and have a lower chance of surviving the winter. However, the positive association between stress metabolite concentration and survival probability has its limit (McEwen and Wingfield, 2003); by late March, we documented a curvilinear relationship between stress metabolite concentrations and survival probability, where animals excreting either low or high concentrations of stress metabolites were predicted to have lower survival probabilities. The reduced survival probability for individuals with high stress metabolite concentrations is likely indicative of chronically stressed calves that are frequently mounting prolonged stress responses throughout the winter months. These findings suggest that fecal stress metabolites and urinary nutritional restriction measures are related to calf survival during a critical point in their first year of life, but these relationships may shift during the prolonged impact of winter tick infestation and winter conditions.

### Management implications

Our study provides previously unavailable perspectives for moose population management, which could help mitigate the impact of winter ticks. Many of the variables in our explanatory models of stress metabolites and nutritional restriction cannot be managed directly (e.g. snow depth) but can be considered when management plans are developed. Our study results support management options such as preventing locally high moose densities and associated tick epizootics, regulating harvests and maintaining optimal moose habitat across a landscape (rather than in restricted locations; Heal*y et al*., 2018; Debow, 2020; Blouin *et al.*, 2021). Management decisions such as harvest regulations can be informed from winter severity indices, particularly during predictable periods of intense tick engorgement. Habitat management should also consider the importance of lower elevation forests, as these areas help moose calves persist through severe winters until nutrient- and protein-rich forage is available in spring. Finally, with the baseline data collected in this study the non-invasive monitoring of moose calf stress metabolites and UN:C ratios may provide useful metrics for winter survival rate estimates, without requiring the expense of radio-collaring moose calves year after year.

## Supplementary material

Supplementary material is available at *Conservation Physiology* online.

## Funding

This work was supported by the Vermont Fish and Wildlife Department in cooperation with the US Fish and Wildlife Service Division of Wildlife and Sportfish Restoration—Wildlife Restoration Program and Safari Club International [06120FY18501]; the US Department of Agriculture National Institute of Food and Agriculture McIntire-Stennis Program [1002300]; and the University of Vermont’s Rubenstein School of the Environment and Natural Resources’ Rubenstein Graduate Fellowship.

## Supplementary Material

TableS1_coab048
